# Hepatic FDG uptake in patients with NAFLD: An important prognostic factor for cardio-cerebrovascular events?

**DOI:** 10.1007/s12350-015-0380-4

**Published:** 2016-01-04

**Authors:** Aukelien C. Dimitriu-Leen, Arthur J. H. A. Scholte

**Affiliations:** Department of Cardiology, Leiden University Medical Center, Albinusdreef 2, Postal zone 2300 RC, 2333 ZA Leiden, The Netherlands

Non-alcoholic fatty liver disease (NAFLD) encompasses the often benign non-alcoholic fatty liver (NAFL) characterized by hepatic steatosis with or without mild inflammation and the more complicated non-alcoholic steatohepatitis (NASH) with lobular inflammation and hepatocellular ballooning which can be complicated by fibrosis.^1^ Independent associates for the presence of liver fibrosis in patients with NASH are diabetes mellitus (DM), insulin resistance, hypertension, weight gain, and increased serum alanine and aspartate aminotransferase.^2^ NAFLD patients with fibrosis are at increased risk for liver cirrhosis. The estimated risks to develop liver cirrhosis are for patients with NASH and patients with NAFL, 22% and 4%, respectively.^3^

In the last decade, the prevalence of NAFLD has tremendously increased as a result of the world-wide raise of patients with DM and obesity (i.e., metabolic syndrome). This has led to a fivefold increase of NAFLD-related liver transplantation.^4^ Moreover, NAFLD is considered to be the hepatic expression of the metabolic syndrome^5^ with augmented atherogenesis expressed by increased carotid intima-media thickness (CIMT), endothelial dysfunction, arterial stiffness, impaired left ventricular function, and coronary calcification. As a result, patients with NAFLD have an increased risk for cardiovascular (CV) disease and mortality.^6^-^8^

Among others, Adams et al demonstrated that the 10-year survival of patients with NAFLD was significantly lower compared with the general population (77 vs 87%, *P*[log-rank] = 0.005), due to higher frequency of fatal CV disease and malignancy.^8^ In addition, Targher et al demonstrated that the presence of NAFLD in asymptomatic patients with DM type 2 was independently associated with an increased risk for myocardial infarction, coronary revascularization procedures, ischemic stroke, and/or CV death (odds ratio 1.84, 95% CI 1.4;2.1, *P* < 0.001).^6^

Currently, the diagnostic reference standard to diagnose NAFLD is a liver biopsy.^9^ However, in an asymptomatic population this invasive technique is not practical as a screening method and not without hazards. Therefore, as an alternative technique, positron emission tomography (PET)/computed tomography (CT) can be used to detect hepatic inflammation by means of the glucose tracer fluorine-18 fluoro-2-deoxyglucose (18F-FDG). 18F-FDG visualizes the importance and utilization of glucose (metabolic activity) of the cells and is expected to be higher in inflammatory cells.^10^ The major drawback of this method is that 18F-FDG PET/CT cannot differentiate between hepatic histologic subtypes.

Results of studies evaluating the hepatic uptake of 18F-FDG measured with PET or PET/CT in NAFLD patients are controversial.^11^-^13^ Abikhzer et al demonstrated in patients with hepatic steatosis a small global decrease in hepatic metabolic activity corrected for lean body mass in comparison with controls.^12^ However, there was no difference when the hepatic standard uptake value (SUV) of 18F-FDG was corrected for body weight. In addition, Lin et al demonstrated a significantly negative correlation in the degree of fatty liver and the maximum hepatic SUV of 18F-FDG on PET.^11^ In contrast, Bural et al showed higher maximum hepatic SUVs on PET in subjects with diffuse hepatic steatosis compared to those in the control group.^13^ A part of the differences in results of the hepatic SUV of 18F-FDG in patients with NAFLD can be explained by the fact that some studies did not take into account lean body mass, glucose levels, and 18F-FDG dose.

Recently, Hong et al demonstrated in 331 asymptomatic men with NAFLD a significantly increased mean hepatic 18F-FDG SUV of 2.40 ± 0.25 in comparison with a mean hepatic 18F-FDG SUV of 2.28 ± 0.26 in 349 controls. In addition, the increased uptake was closely correlated with serum γ-glutamyl transpeptidase and triglycerides, markers for hepatic inflammation and injury.^14^

In this issue of the journal, the same group addressed the role of hepatic 18F-FDG uptake for predicting future CV and cardio-cerebrovascular events and evaluated its prognostic value in comparison with other CV risk factors including the Framingham risk score and CIMT.^15^ In a recent study, 815 asymptomatic participants underwent a health screening program that consisted of 18F-FDG PET/CT, abdominal ultrasonography, and CIMT measurements. The primary endpoint consisted of CV events including myocardial infarction, coronary intervention for significant coronary stenosis, and angina requiring an emergency room visit with demonstration of significant coronary stenosis. Additional analysis evaluated the combined endpoint cerebrovascular (consisting of stroke, transient ischemic attacks, and deaths) and cardiovascular events. Moon et al demonstrated that the only independent factor for future CV events in this asymptomatic population was the combination of high hepatic FDG uptake and NAFLD (determined by abdominal sonography and questionnaire about alcohol intake). This remained after including cerebrovascular events. In the NAFLD subgroup, high hepatic FDG uptake and male were independently associated with future CV events. For the combined endpoint cardio-cerebrovascular events, only high hepatic FDG uptake was an independent factor in the NAFLD subgroup. However, the conclusions of the authors should be placed in a broader perspective. First, the study results might not be representative for the general population since the study population comprised a high percentage of male (>90%). Second, there were some small differences in the procedure of patients’ preparation for 18F-FDG PET/CT in comparison with the guidelines which might influence the implementation.^16^ The cut-off value of blood glucose levels at the time of FDG injection was higher (<200 mg/dl instead of an upper plasma level range between 126 and 150 mg/dl which is nowadays recommended in a research population). Third, evaluation of CV and cardio-cerebrovascular events in an asymptomatic cohort is challenging since event rates are low. In line with expected, the CV event rates were indeed low, in the control group as well as in the NAFLD group, 0.7% (3/421) and 1.5% (6/394), respectively. Therefore, conclusions on differences in CV event rates between patients with and without NAFLD are based on an absolute difference of 3 events. In the additional analysis after inclusion of cerebrovascular events, the absolute difference in events between the groups was even smaller, only 2 events (1.2% (5/421) vs 1.8% (7/394), respectively). Although independently associated in multivariate analyses, the additive value of screening asymptomatic patients for NAFLD in combination with increased hepatic 18F-FDG SUV on PET/CT on top of traditional risk scores is limited given the small absolute numbers. As well, the radiation exposure of PET/CT should be taken into account. The effective dose from 18F-FDG in adults is about 7 mSv for an administrated activity of 370 MBq.^17^ On top, the CT-related radiation dose should be added. This radiation dose differs from patient to patient and ranges from 1 up to 20 mSv, depending on the type of scanner and body mass index. In conclusion, we have to be aware that patients with NAFLD and no cardio-cerebrovascular complaints are at increased risk for these events. However, since we realize that a liver biopsy is not the ideal screening tool, determining hepatic FDG uptake on PET/CT scan could be a good non-invasive alternative to estimate the risk of these patients but needs more data and convincing proof.

## References

[1] Kleiner DE, Brunt EM, Van Natta M, Behling C, Contos MJ, Cummings OW (2005). Design and validation of a histological scoring system for nonalcoholic fatty liver disease. Hepatology.

[2] Angulo P, Keach JC, Batts KP, Lindor KD (1999). Independent predictors of liver fibrosis in patients with nonalcoholic steatohepatitis. Hepatology.

[3] Matteoni CA, Younossi ZM, Gramlich T, Boparai N, Liu YC, McCullough AJ (1999). Nonalcoholic fatty liver disease: A spectrum of clinical and pathological severity. Gastroenterology.

[4] Agopian VG, Kaldas FM, Hong JC, Whittaker M, Holt C, Rana A (2012). Liver transplantation for nonalcoholic steatohepatitis: The new epidemic. Ann Surg.

[5] Khashab MA, Liangpunsakul S, Chalasani N (2008). Nonalcoholic fatty liver disease as a component of the metabolic syndrome. Curr Gastroenterol Rep.

[6] Targher G, Bertolini L, Poli F, Rodella S, Scala L, Tessari R (2005). Nonalcoholic fatty liver disease and risk of future cardiovascular events among type 2 diabetic patients. Diabetes.

[7] Hamaguchi M, Kojima T, Takeda N, Nagata C, Takeda J, Sarui H (2007). Nonalcoholic fatty liver disease is a novel predictor of cardiovascular disease. World J Gastroenterol.

[8] Adams LA, Lymp JF, St Sauver J, Sanderson SO, Lindor KD, Feldstein A (2005). The natural history of nonalcoholic fatty liver disease: A population-based cohort study. Gastroenterology.

[9] Sumida Y, Nakajima A, Itoh Y (2014). Limitations of liver biopsy and non-invasive diagnostic tests for the diagnosis of nonalcoholic fatty liver disease/nonalcoholic steatohepatitis. World J Gastroenterol.

[10] Keramida G, Potts J, Bush J, Verma S, Dizdarevic S, Peters AM (2014). Accumulation of (18)F-FDG in the liver in hepatic steatosis. Am J Roentgenol.

[11] Lin CY, Lin WY, Lin CC, Shih CM, Jeng LB, Kao CH (2011). The negative impact of fatty liver on maximum standard uptake value of liver on FDG PET. Clin Imaging.

[12] Abikhzer G, Alabed YZ, Azoulay L, Assayag J, Rush C (2011). Altered hepatic metabolic activity in patients with hepatic steatosis on FDG PET/CT. Am J Roentgenol.

[13] Bural GG, Torigian DA, Burke A, Houseni M, Alkhawaldeh K, Cucchiara A (2010). Quantitative assessment of the hepatic metabolic volume product in patients with diffuse hepatic steatosis and normal controls through use of FDG-PET and MR imaging: A novel concept. Mol Imaging Biol.

[14] Hong SP, Noh TS, Moon SH, Cho YS, Lee EJ, Choi JY (2014). Hepatic glucose uptake is increased in association with elevated serum gamma-glutamyl transpeptidase and triglyceride. Dig Dis Sci.

[15] Moon SH, Hong S-P, Cho YS, Noh TS, Choi JY, Kim B-T, Lee K-H (2014). Hepatic FDG uptake is associated with future cardiovascular events in asymptomatic individuals with non-alcoholic fatty liver disease. J Nucl Cardiol.

[16] Boellaard R, Delgado-Bolton R, Oyen WJ, Giammarile F, Tatsch K, Eschner W (2015). FDG PET/CT: EANM procedure guidelines for tumour imaging: Version 2.0. Eur J Nucl Med Mol Imaging.

[17] ICRP (2008). Radiation dose to patients from radiopharmaceuticals. Addendum 3 to ICRP Publication 53. ICRP Publication 106. Approved by the Commission in October 2007. Ann ICRP.

